# Predictive value of C-reactive Protein for antidepressant treatment response in patients with major depressive disorder

**DOI:** 10.1192/j.eurpsy.2026.11806

**Published:** 2026-07-31

**Authors:** K. J. Lee, H. Kim

**Affiliations:** Psychiatry, Inje Univ. Ilsanpaik Hospital, Goyang-si, Korea, Republic Of

## Abstract

**Introduction:**

Recently, 0.15pt?>it has been found that immune and inflammatory responses are deeply related to major depressive disorder(MDD) and drug treatment, and research on inflammatory factors as predictors of treatment response has been actively conducted. Results have also been published that increased levels of C-reactive protein (CRP) are associated with the occurrence of depression, and recent studies have found that the treatment response differs according to the CRP baseline level.

**Objectives:**

This study aimed to evaluate whether baseline levels of CRP are significantly associated with treatment response to selective serotonin reuptake inhibitors(SSRIs) in patients with MDD.

**Methods:**

Among 60 outpatients diagnosed with MDD according to DSM-5 criteria, 38 patients completed the study. Participants were treated with escitalopram for 8 weeks. Serum CRP levels were measured prior to medication, and depressive symptoms were assessed using the Hamilton Depression Rating Scale, 17-item version (HAMD-17) and the Patient Health Questionnaire-9 (PHQ-9) at baseline and week 8. Pearson correlation and multivariate logistic regression analyses were conducted to examine the relationship between baseline CRP levels and treatment response.

**Results:**

The mean age of the 38 subjects with MDD was 60.55 ± 15.16 years. Among them, fifteen were male and twenty-three were female. The mean daily dose of the antidepressant escitalopram was 11.05 ± 4.37 mg, and the mean level of CRP was 0.12 ± 0.12 mg/dL. Depressive symptoms significantly improved after treatment (Table 1). However, baseline CRP levels were not significantly correlated with symptom changes (Table 2). CRP was not a significant predictor of treatment response, and its predictive validity was limited (Table 3).

**Image 1:**
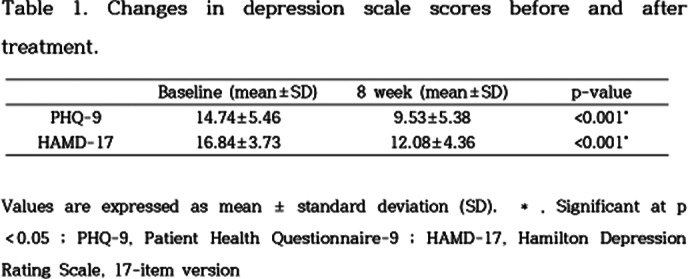

**Image 2:**
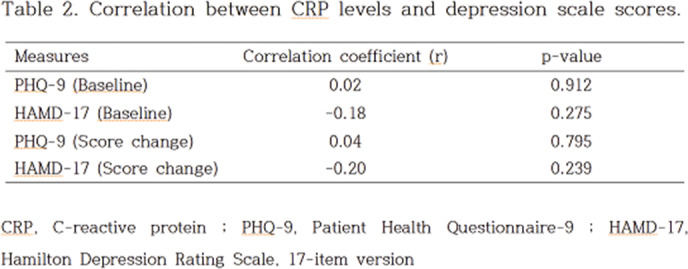

**Image 3:**
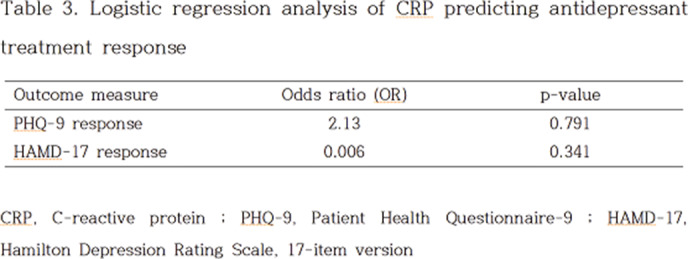

**Conclusions:**

This study suggests that CRP may have limited utility as a biological predictor of SSRI treatment response in patients with MDD. Future research should incorporate larger sample sizes and multimodal biomarker-based predictive models.

**Disclosure of Interest:**

None Declared

